# Sonic Hedgehog Signaling Agonist (SAG) Triggers BDNF Secretion and Promotes the Maturation of GABAergic Networks in the Postnatal Rat Hippocampus

**DOI:** 10.3389/fncel.2020.00098

**Published:** 2020-04-23

**Authors:** Quentin Delmotte, Diabe Diabira, Yasmine Belaidouni, Mira Hamze, Marine Kochmann, Aurélie Montheil, Jean-Luc Gaiarsa, Christophe Porcher, Yesser H. Belgacem

**Affiliations:** ^1^Aix-Marseille Univ, Marseille, France; ^2^INSERM (Institut National de la Santé et de la Recherche Médicale) Unité 1249, Marseille, France; ^3^INMED (Institut de Neurobiologie de la Méditerranée), Parc Scientifique de Luminy, Marseille, France; ^4^Institut des Neurosciences de La Timone, Marseille, France

**Keywords:** GABA, sonic hedgehog, GDP, hippocampus, BDNF

## Abstract

Sonic hedgehog (Shh) signaling plays critical roles during early central nervous system development, such as neural cell proliferation, patterning of the neural tube and neuronal differentiation. While Shh signaling is still present in the postnatal brain, the roles it may play are, however, largely unknown. In particular, Shh signaling components are found at the synaptic junction in the maturing hippocampus during the first two postnatal weeks. This period is characterized by the presence of ongoing spontaneous synaptic activity at the cellular and network levels thought to play important roles in the onset of neuronal circuit formation and synaptic plasticity. Here, we demonstrate that non-canonical Shh signaling increases the frequency of the synchronized electrical activity called Giant Depolarizing Potentials (GDP) and enhances spontaneous GABA post-synaptic currents in the rodent hippocampus during the early postnatal period. This effect is mediated specifically through the Shh co-receptor Smoothened *via* intracellular Ca^2+^ signal and the activation of the BDNF-TrkB signaling pathway. Given the importance of these spontaneous events on neuronal network maturation and refinement, this study opens new perspectives for Shh signaling on the control of early stages of postnatal brain maturation and physiology.

## Introduction

Sonic Hedgehog (Shh) signaling is a highly conserved pathway among invertebrates and vertebrates (Kumar et al., 1996), mostly known for its roles during embryonic central nervous system development on neural cell proliferation, neural progenitor specification and neuronal differentiation (Ruat et al., 2012, 2014; Briscoe and Thérond, 2013; Belgacem et al., 2016). Despite its known role as a mitogen, more recent studies have shown other functions of Shh affecting later phases of central nervous system development. For instance, Shh regulates axonal guidance (Yam et al., 2009; Makihara et al., 2018; Peng et al., 2018), axonal elongation (Yao et al., 2015) and, in cultured hippocampal neurons, it regulates glutamatergic synaptic size and function (Mitchell et al., 2012). Shh binds to its receptor Patched 1 (Ptch1), which releases the constitutive inhibition it exerts on Shh-coreceptor Smoothened (Smo), a member of the G protein-coupled receptor family. Smo, in turn, activates the main downstream canonical effectors of Shh signaling, the Glioma-associated oncogen (Gli) family of transcription factors (Ruat et al., 2012; Briscoe and Thérond, 2013; Ruat et al., 2014; Belgacem et al., 2016). Shh signaling also initiates several non-canonical, Gli-independent signaling pathways, including the GTPase RhoA (Polizio et al., 2011) or Rac1 (Sasaki et al., 2010), Src family kinases (Yam et al., 2009) and the AMPK axis (Teperino et al., 2012). Moreover, interactions between Smo signaling and Ca^2+^ activity have been identified in hippocampal neurons (Feng et al., 2016) and the spinal cord for the specification of the GABAergic phenotype (Belgacem and Borodinsky, 2011; Belgacem et al., 2016). Interestingly, Ptch1 and Smo have been described in immature and adult rat hippocampal neurons. In postnatal neurons, Ptch1 and Smo are co-localized in the processes and growth cones (Petralia et al., 2011), whereas in adult neurons Shh receptors are present in the post-synaptic sides in several hippocampal areas including CA3 and CA1 pyramidal neurons (Petralia et al., 2011) and the granule cells of the dentate gyrus (Charytoniuk et al., 2002). Moreover, functional analysis of cultured adult hippocampal neurons revealed that Shh signaling regulated the structure and the electrophysiological properties of presynaptic terminals (Mitchell et al., 2012). Besides, a recent study demonstrated that Shh is released by hippocampal neurons in an activity-dependent manner (Su et al., 2017). Altogether, these data raise the question of a new role for Shh acting as a neurotrophic factor during this particular developmental stage of synapse formation and establishment of neuronal networks (Charytoniuk et al., 2002; Traiffort et al., 2010; Petralia et al., 2011, 2012; Yao et al., 2016).

The perinatal period corresponds to a peak in synaptogenesis and the emergence of spontaneous electrical activity important for the formation of functional GABAergic synapses (Colin-Le Brun et al., 2004; Griguoli and Cherubini, 2017). In the developing rodent hippocampus, ongoing synaptic activity occurs at the cellular and network levels and is characterized by a developmental sequence with GABAergic synapses formed before glutamatergic synapses (Ben-Ari et al., 2007). Consequently, proper GABAergic synapse formation during brain development is instrumental and alterations in GABA connectivity may lead to neurodevelopmental disorders including Autism Spectrum Disorder and epilepsy (Kuzirian and Paradis, 2011; Ben-Ari et al., 2012; Canitano and Pallagrosi, 2017). Parallel to this sequential process of GABA and glutamate synapse formation, in the developing hippocampus, spontaneous network activity is characterized by the presence of Giant Depolarizing Potentials (GDP; Ben-Ari et al., 1989; Griguoli and Cherubini, 2017). These spontaneous network events are generated by a synchronized discharge of glutamatergic and GABAergic inputs (Ben-Ari et al., 1989, 2007) and are of importance in the formation and stabilization of neuronal assemblies through synaptic plasticity facilitation (Mohajerani et al., 2007; Griguoli and Cherubini, 2017). Interestingly, Shh signaling modulates early spontaneous electrical activity in the embryonic spinal cord, which is crucial for spinal neuron differentiation and GABAergic phenotype homeostatic specification (Belgacem and Borodinsky, 2011, 2015; Belgacem et al., 2016).

In the present study, we discover that activating Smo-dependent Shh signaling leads to an increase in spontaneous GABAergic synaptic currents and to a long-lasting potentiation in GDP frequency. Furthermore, we show that this effect requires an intracellular calcium signal, and the secretion of BDNF followed by the activation of the subsequent TrkB signaling pathway. The findings also suggest the existence of a critical period for Shh-Smo signaling modulation of spontaneous GABAergic synaptic activity in the immature hippocampus. This study describes a novel function for the Shh pathway during postnatal development on early synaptic and network activities.

## Materials and Methods

### Animals

All animal experiments were carried out according to the guidelines set by the INSERM animal welfare through the local committee and the European Communities Council Directive of 24 November 1986 (86/609/EEC). Experiments were performed on male and female Wistar rats (Janvier Labs) from postnatal day 1 (P1) to 15 (P15). Animals were housed in a temperature-controlled environment with a 12-h light/dark cycle at 22-24°C and free access to food and water.

### Drugs

The following reagents were used from the indicated source: Smo agonist (SAG) and Cyclopamine from Tocris (Bristol, UK). 1,2,3,4-Tetrahydro-6-nitro-2,3-dioxo-benzo[f]quinoxaline-7-sulfonamide (NBQX), D-2-amino-5-phosphovaleric acid (D-APV) from the Molecular, Cellular, and Genomic Neuroscience Research Branch (MCGNRB) of the National Institute of Mental Health (NIMH, Bethesda, MD, USA). 1.2- bis (2-Aminophenoxy)ethane-N,N,N′,N′-tetraacetic acid (BAPTA) and BAPTA-AM from Sigma (St. Louis, MN, USA). TrkB- and TrkC-IgG were purchased from R&D system (Minneapolis, MN, USA). Tetrodotoxin (TTX) was purchased from Abcam (Bristol, UK).

### Hippocampal Slices Preparation

Brains were removed and immersed into ice-cold (2-4°C) artificial cerebrospinal fluid (ACSF) with the following composition (in mM): 126 NaCl, 3.5 KCl, 2 CaCl_2_, 1.3 MgCl_2_, 1.2 NaH_2_PO_4_, 25 NaHCO_3_ and 11 glucose, pH 7.4 equilibrated with 95% O_2_ and 5% CO_2_. Hippocampal slices (350 μm thick) were cut with a McIlwain tissue chopper (Campden Instruments Limited) and kept in oxygenated (95% O_2_ and 5% CO_2_) ACSF at room temperature (25°C) for at least 1 h before recording. Slices were then transferred to a submerged recording chamber perfused with oxygenated (95% O_2_ and 5% CO_2_) ACSF (3 ml/min) at 34°C.

### Electrophysiological Recordings

Extracellular field potentials were recorded by using tungsten wire electrodes (diameter: 50 μm, California Fine Wire, Grover Beach, CA, USA) and a low-noise multichannel DAM-8A amplifiers (WPI, GB; low filter: 0.1 Hz; high filter: 3 kHz; 1,000×) located in the stratum pyramidal of the CA3 region. Whole-cell patch-clamp recordings of CA3 pyramidal neurons were performed with an Axopatch 200B amplifier (Axon Instruments, Foster City, CA, USA). For sGABA-IPSCs-only recording, microelectrodes (4–8 MΩ) were filled with the following solution (in mM): CsCl (110), K-gluconate (30), HEPES (10); EGTA (1.1), CaCl_2_ (0.1), MgATP (4), NaGTP (0.3). With this solution, the GABA_A_ receptor-mediated synaptic currents reversed at a membrane potential of 0 mV. GABA spontaneous activity was isolated using ionotropic glutamatergic receptor antagonists NBQX (5 μM) and D-APV (40 μM) while membrane potential was held at −70 mV. Smo-agonist SAG was applied 15 min at the indicated concentrations and in presence of indicated drugs: cyclopamine (2 μM), BDNF scavenger TrkB-IgG (1 μg/ml) or TrkC-IgG (1 μg/ml). To block intracellular calcium signaling in the indicated experiment, calcium-chelator BAPTA (10 mM) was added in microelectrodes in addition to the above intrapipette solution.

For the simultaneous recording of spontaneous GABA inhibitory (sGABA-IPSCs) and glutamate excitatory (sAMPA-EPSCs) post-synaptic currents, glass recording electrodes (4–7 MΩ) were filled with a solution containing (in mM): 100 KGluconate, 13 KCl, 10 N-2-hydroxyethyl piperazine-N′-2-ethane sulfonic acid (HEPES), 1.1 ethylene glycol-bis (β-aminoethyl ether)-N,N,N′,N′-tetra-acetic acid (EGTA), 0.1 CaCl_2_, 4 MgATP, and 0.3 NaGTP. The pH of the intracellular solution was adjusted to 7.2 and the osmolality to 280 mOsmol l^−1^. With this solution, GABA-IPSCs reversed at −70 mV. GABA-IPSCs and AMPA-EPSCs were simultaneously recorded at a holding potential of −45 mV.

The signals were digitized using a Digidata1440A converter (Axon Instruments, San Jose, CA, USA). Axoscope software version 8.1 (Axon Instruments, San Jose, CA, USA) or pCLAMP1 0.0.1.10 (Axon Instruments, San Jose, CA, USA) and MiniAnalysis 6.03 (Synaptosoft, Decatur, CA, USA) programs were used for acquisition and analysis. Series resistance (R_s_), membrane capacitance (Cm) and input resistance (R_i_) were determined by an online fitting analysis of the transient currents in response to a 5-mV pulse with Acquis 4.0 software (Bio-logic, Orsay, France). Criteria for accepting a recording included a resting potential < −55 mV, R_i_ > 400MΩ, R_s_ < 25MΩ and cells exhibiting more than 20% change in series resistance were excluded from the analysis.

### Sonic Hedghehog Protein (Shh) Immunoassay

Hippocampal tissues from rats at the indicated age were homogenized in RIPA buffer (150 mM NaCl, 1% Triton X100, 0.1% SDS, 50 mM Tris HCl, pH 8, containing protease inhibitors (Complete Mini; Roche). Lysates were centrifuged (5000 *g* for 5 min at 4°C). Loading was 200 μg of protein as determined using a modified Bradford reaction (BioRad Laboratories). Quantification of Shh was performed with Rat Shh ELISA Kit (FineTest, Wuhan Fine Biotech Company Limited, China) in the concentrated solutions following the manufacturer’s protocol. Experiments and analyses were done blindly.

### Primary Cultures of Rat Hippocampal Neurons

Neurons from 18-day-old rat embryos were dissected and dissociated using 0.05% Trypsin (Gibco) and plated at a density of 70,000 cells cm^−2^ in minimal essential medium (MEM) supplemented with 10% NU serum (BD Biosciences, Le Pont de Claix, France), 0.45% glucose, 1 mM sodium pyruvate (Invitrogen), 2 mM glutamine, 15 mM HEPES Buffer (Invitrogen) and 10 IU ml^−1^ penicillin-streptomycin (Invitrogen) as previously described (Kaech and Banker, 2006). On days 7, 10 and 13 of culture incubation (DIV, days *in vitro*), half of the medium was changed to MEM with 2% B27 supplement (Invitrogen). For electrophysiology, neuronal cultures were plated on coverslips placed in 35-mm culture dishes. Twelve hours before plating, dishes with coverslips were coated with polyethyleneimine (5 mg/ml).

### Electrophysiological Recordings in Neuronal Cell Culture

Electrophysiological recordings from neurons were performed at 14 DIV. Neurons were continuously perfused with an extracellular solution containing the following (in mM): 150 NaCl, 2.5 KCl, 2.5 HEPES, 20 D-glucose, 2.0 CaCl_2_, 2.0 MgCl_2_, 0.01 CNQX, 0.4 D-APV pH 7.4, at 1 ml/min. Recording electrodes (4–6 MΩ) were filled with a solution containing the following (in mM): 140 CsCl, 10 HEPES, 2.5 MgCl_2_, 4 Na-ATP, 0.4 Na-GTP, 10 sodium phosphocreatine, 0.6 EGTA, pH 7.2, and with calcium-chelator BAPTA for indicated experiment. Smo-agonist SAG was applied in the bath at 100 nM for 10 min. Recordings were made using an Axopatch-200A amplifier and pCLAMP acquisition software (Molecular Devices). Series resistance was compensated electronically. Data were low-pass filtered at 2 kHz and acquired at 10 kHz. Spontaneous GABA postsynaptic currents were analyzed blindly using Mini Analysis software (Synaptosoft). All experiments were performed at 22–24°C.

### Surface BDNF-mCherry Immunofluorescence Analysis

The procedure was similar to that previously described (Kuczewski et al., 2008b; Porcher et al., 2011). Briefly, hippocampal neurons (14 DIV) were transfected with cDNAs encoding the BDNF-mCherry using CombiMag Transfection Reagent (OZ Biosciences; Buerli et al., 2007). The cultures were incubated with TTX (0.5 μM) for 1 h in the presence or absence of cyclopamine (2 μM), followed by NBQX (5 μM) and D-APV (40 μM) for 10 min. Cell cultures were then stimulated for 10 min with SAG (100 nM). The omission of SAG or incubation with KCl (50 mM) was used as negative and positive controls respectively. Live cultures were incubated at 10°C for 1 h in the presence of an anti-DsRed rabbit antibody (10 μg/ml; BioVision). Immediately after treatment, cultures were fixed for 30 min with 4% PFA at 4°C. After fixation, cultures were permeabilized and co-incubated overnight with mouse anti-MAP2 (1:2,000). Cells were exposed to a saturating concentration (10 μg/ml) of FITC-conjugated anti-rabbit IgG (Invitrogen) and Cy5-conjugated anti-mouse IgG (Millipore) for 1.5 h. Quantification was performed using ImageJ software (NIH, Bethesda, MD, USA^1^). The ratio of surface-bound BDNF-mCherry-FITC to total BDNF-mCherry was estimated as the ratio of the area of co-localized FITC and BDNF-mCherry/total area of BDNF-mCherry and expressed as the ratio of co-localized signals. Images were acquired using a laser scanning confocal microscope (Zeiss LSM 510 Meta) with a 40× oil-immersion objective.

### Phospho-CREB Activation and Immunocytochemistry

The procedure was similar to that previously described (Kuczewski et al., 2008b; Fiorentino et al., 2009). Briefly, 1 day before stimulation (at 13 DIV) one-half of the culture medium was changed to MEM with 2% B27 supplement. The cultures were then stimulated with SAG (100 nM) for 10 min in the absence or presence of different drugs, as described in the results section and figure legends. After stimulation, neurons were fixed, permeabilized and co-incubated overnight with mouse anti-CREB (1:1,000; Cell Signaling Technology, catalog no #9104), rabbit anti-phospho-CREB (pCREB, 1:1,000; Cell Signaling Technology, no #9198) and with chicken anti-MAP2 (1:4,000, Sigma, no AB5543) antibodies. Immunoreactivity for pCREB, CREB and MAP2 were detected with secondary antibodies coupled to rabbit-Alexa 488 (1:1,000; Cell Signaling Technology, no #4412), mouse-Cy3 (1:1,000; Merck Millipore, no AP130C) and chicken-Alexa 647 (1:1,000; Merck Millipore, no AP194SA6) respectively. All procedures were performed in a phosphate-free solution containing 140 mM NaCl, 5 mM KCl and 10 mM HEPES-Na, pH 7.4. The acquisition (Zeiss LSM 510) of A488 (pCREB), Cy3 (CREB) and then Cy5 (MAP2) was sequential to avoid overlap of excitation and emission of fluorescence. The optical sections were digitized (1,024 × 1,024 pixels) and processed using ImageJ software.

### Real-Time qRT-PCR

RNA was isolated and quantified by reading the absorbance at 260 nm (NanoPhotometer, IMPLEN) using an RNeasy kit (Qiagen), then converted to cDNA using 1 μg RNA and a QuantiTect Reverse Transcription kit (Qiagen) according to the manufacturer’s instructions. PCR was carried out with the LightCycler 480 SYBR Green I Master (Roche Applied Science) with 1 μl cDNA using the following oligonucleotides (QuantiTect Primer Assay, Qiagen): Smo: QT00190183; Ptch1: QT01579669; Gli1: QT01290324 and glyceraldehyde-3-phosphate dehydrogenase (GAPDH): QT001199633. Relative mRNA values were calculated using the LC480 software and GAPDH as the housekeeping gene. The minimal sample size for each reported group analyzed using qRT-PCR is three biological replicates.

### Statistics

To ensure the consistency and reproducibility of our results, we conducted repeated trials (referred to as n) prepared from at least three independent experiments or animals (referred to as N) for each experimental condition. No statistical methods were used to predetermine sample sizes, but our sample sizes correspond to those reported in previous publications (Guimond et al., 2014; Riffault et al., 2014; Dumon et al., 2019). A Shapiro–Wilk test was used to verify if the data were normally or not normally distributed. If not stated otherwise, statistics are presented as the median only for non-normally distributed data. For data displaying non-normal distribution, Mann-Whitney U-test was used for comparison between two independent groups and Wilcoxon matched-pairs signed-rank test to analyze differences within one group across conditions. Statistical analyses and assessment of normal or non-normal distribution (Shapiro–Wilk test) were performed with GraphPad Prism (GraphPad software 5.01).

## Results

### SAG Enhances GDP Frequency in a Dose-Dependent Manner During a Critical Temporal Window

**Figure 1:**
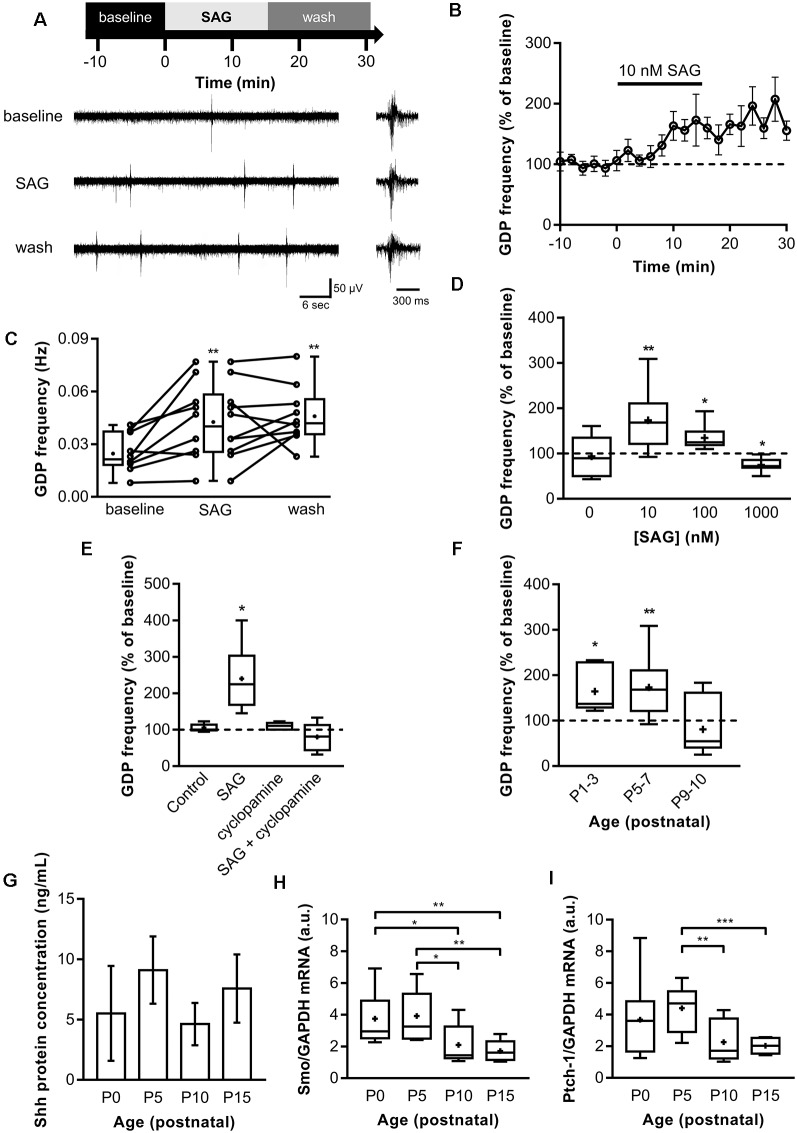
Shh-coreceptor Smoothened (Smo) signaling modulates Giant Depolarizing Potentials (GDP) frequency. **(A)** Extracellular field recordings of GDP at P5 to P7 in the CA3 pyramidal layer during 10-min control baseline (baseline), 15-min application of 10 nM Smo-agonist (SAG) and 15-min of wash. GDP are shown at an expanded time scale on the right. **(B)** Time course of mean GDP frequency ± SEM (2-min bin) normalized to average frequency during baseline period preceding SAG application. **(C)** Box plot and individual data points show GDP frequency in baseline (10-min period before SAG application), SAG (last 10-min of SAG application) and wash. Median frequency: 0.021 Hz during control baseline and 0.04 Hz during SAG; *p* = 0.005, *N* = 6 animals, *n* = 10 slices; and 0.042 Hz during wash; *p* = 0.009 vs. control baseline, *N* = 6, *n* = 10; Wilcoxon test. **(D)** SAG effect on GDP frequency is dose-dependent. Box plot shows median GDP frequency in control condition or during SAG application at different concentrations, normalized to GDP frequency during baseline. Median values: 100% for control (0 nM); *p* = 0.84, *N* = 3, *n* = 6; 168% for 10 nM SAG compared to control baseline; *p* = 0.0059, *N* = 5, *n* = 10: 124.6% for 100 nM SAG; *p* = 0.03, *N* = 4, *n* = 6; and 72% for 1 μM SAG;* p* = 0.015, *N* = 4, *n* = 7; Wilcoxon test. **(E)** Box plot shows the effect on GDP frequency of the application of carrier only (0.1% ethanol, Control), 10 nM SAG in 0.1% ethanol (SAG), 2 μM cyclopamine preincubated 30 min (cyclopamine in 0.1% ethanol), or SAG in the presence of 2 μM cyclopamine preincubated 30 min before (SAG + cyclopamine in 0.1% ethanol). Median values: 100.2% for Control; *p* = 0.15, *N* = 4, *n* = 9; 224.6% for SAG; *p* = 0.03 compared to baseline period, *N* = 6, *n* = 6; 110% for cyclopamine alone; *p* = 0.25, *N* = 3, *n* = 6; and 81.4% for SAG + cyclopamine; *p* = 0.46, *N* = 6, *n* = 6; Wilcoxon test. **(F)** SAG effect is developmentally regulated. Box plot shows the effect of 10 nM SAG application on GDP frequency at different postnatal time points. Median values: 136.8% at P1-3;* p* = 0.03, *N* = 4, *n* = 6; 168.1% at P5-7; *p* = 0.0059, *N* = 5, *n* = 10; and 54.84% at P9-10; *p* = 0.45, *N* = 3, *n* = 7; Wilcoxon test. **(G)** Shh protein level remains stable during the first two postnatal weeks. Box plot shows median Shh protein concentration measured by ELISA between P0 and P15 in hippocampus lysates. Median values: 4.53 ng/ml at P0, *n* = 4; 9.5 ng/ml at P5, *n* = 3; 5.3 ng/ml at P10, *n* = 3; and 6.73 ng/ml at P15, *n* = 3; *p* > 0.05, Mann–Whitney *U* test. **(H)** Smoothened and **(I)** Patched-1 mRNA level are developmentally regulated. Box plots show Shh pathway components mRNA levels in hippocampus at different ages. Median values: For Smo mRNA level: 2.95 a.u. at P0, *N* = 9; 3.26 a.u. at P5, *N* = 10; 1.44 a.u. at P10, *N* = 9; and 1.61 a.u. at P15, *N* = 8; *p* = 0.03 for P0 vs. P10; *p* = 0.0025 for P0 vs. P15; *p* = 0.017 for P5 vs. P10; and *p* = 0.0014 for P5 vs. P15; Mann–Whitney *U* test. For Ptch-1 mRNA level: 3.61 a.u. at P0, *N* = 9; 4.70 a.u. at P5, *N* = 10; 1.71 a.u. at P10, *N* = 9; and 2.03 a.u. at P15, *N* = 8; *p* = 0.004 for P5 vs. P10; *p* = 0.0005 for P5 vs. P15; Mann–Whitney *U* test. *p < 0.05, **p < 0.01, ***p < 0.001.

Spontaneous electrical activity is important to promote specific steps of central nervous system development and is therefore found during specific time windows in the early stages of postnatal development. To assess a potential role of Shh signaling in ongoing spontaneous synaptic activity, field potential recordings were made on acute hippocampal slices from P5 to P7 while bath applying the Shh signaling agonist (SAG; Chen et al., 2002b) at concentrations of 10 nM, 100 nM and 1 μM (Belgacem and Borodinsky, 2011, 2015; Feng et al., 2016) for 15 min. In control hippocampi, the GDP median duration is 200–300 ms with a median frequency of 0.021 Hz (Figures 1A–C). During the application of 10 nM SAG, the median GDP frequency is increased to 0.04 Hz and stays high during wash at 0.042 Hz (Figures 1A–C), thus suggesting a long-lasting potentiation effect of SAG on GDPs. Furthermore, our results indicate that the effect of SAG on GDP frequency is dose-dependent. Indeed, 10 and 100 nM SAG increased GDP frequency (Figure 1D). On the other hand, 1 μM of SAG application decreased GDP frequency (Figure 1D). This effect agrees with previous *in vitro* studies on NIH 3T3 cell cultures that have shown that high concentrations of SAG (i.e., above 1 μM) induce less Shh signaling activation than lower doses in the range of 100 nM (Chen et al., 2002b). To ensure that the action of SAG was specific to the Smo signaling pathway, we pre-incubated slices with cyclopamine, a competitive antagonist of Smo that binds to the same domain as SAG (Chen et al., 2002a; Ruat et al., 2014). We found that treatment with 2 μM cyclopamine (30 min) showed no effect on GDP when compared to baseline activity but prevented SAG-induced increase in GDP frequency (Figure 1E).

GDPs are only present during the first two postnatal weeks in the rodent hippocampus (Khazipov et al., 2004). We next assessed whether SAG signaling modulates GDP during this critical period by activating the Smo receptor with SAG application (10 nM) on acute hippocampal slices from P1 to P10 rats. We found that SAG induces an increase in GDP frequency on acute slices at both P1-P3 and P5-7 rats (Figure 1F). In contrast, at P9-P10, SAG did not affect significantly GDP frequency (Figure 1F).

We next measured Shh protein expression levels by ELISA. Our results show that Shh protein is present in the hippocampus at least during the two first postnatal weeks with no significant variations in the level of Shh proteins between P0 and P15 (Figure 1G). Interestingly, the mRNA levels for the Shh co-receptors, Smo and Ptch1 in the rat hippocampus are relatively stable from P0 to P5 but decrease after P10 (Figures 1H,I).

Altogether, these results indicate that Shh protein is present during this postnatal period and activation of Shh signaling increases GDP frequency in a dose-dependent manner through the co-receptor Smo during a critical period from birth to P7, when the expression of Shh co-receptors is high.

### SAG Increases GABAergic Spontaneous Activity

**Figure 2:**
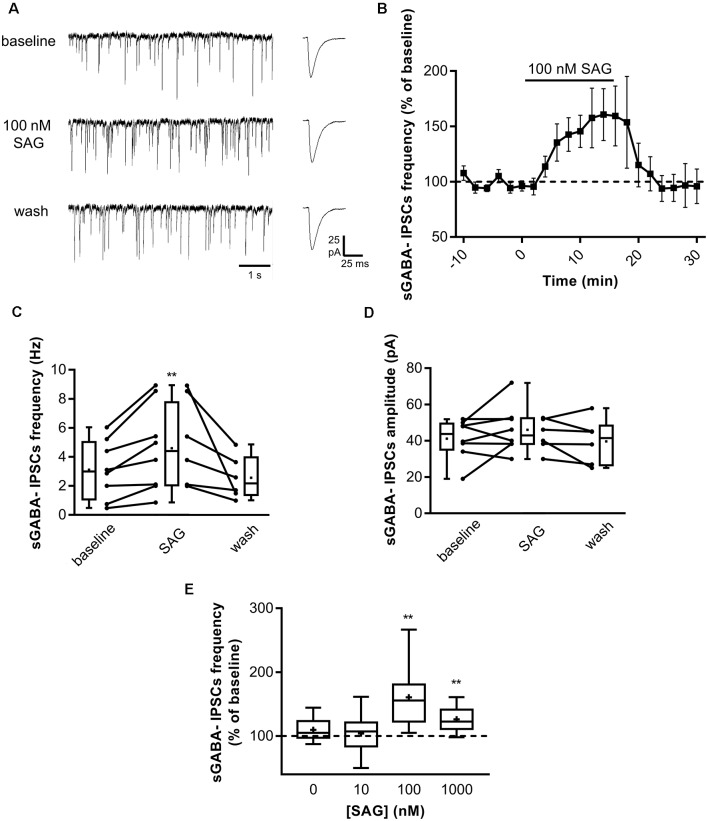
SAG modulates GABAergic spontaneous activity. **(A)** Representative traces of spontaneous GABAergic inhibitory post-synaptic currents (sGABA-IPSCs) during baseline, SAG application (100 nM) and wash. Averaged sGABA-IPSCs (*n* = 20 events) are shown at an expanded time scale on the right. **(B)**) Time course of mean sGABA-IPSCs frequency ± SEM (2-min bin) during SAG application, normalized to baseline period. **(C,D)** Box plots and individual data points show sGABA-IPSCs frequency **(C)** and amplitude **(D)** during baseline, SAG application and wash. Median values: for sGABA-IPSCs frequency: 2.97 Hz for baseline vs. 4.38 Hz during SAG; *p* = 0.007, *N* = 4 animals, *n* = 8 slices; and 2.15 Hz during wash; *p* = 0.06 compared to baseline, *N* = 3, *n* = 6; Wilcoxon test. For sGABA-IPSCs amplitude: 43.08 mV during baseline vs. 43.8 mV during SAG; *p* = 0.37, *N* = 4, *n* = 8; and 41.5 mV during wash; *p* = 0.99 compared to baseline, *N* = 3 *n* = 6; Wilcoxon test. **(E)** SAG effect on sGABA-IPSCs frequency is dose-dependent. Box plot show median sGABA-IPSCs frequency in control condition or during SAG application at different concentrations, normalized to baseline period. Median values: 105.2% for control; *p* = 0.13, *N* = 5, *n* = 12; 107.3% for SAG at 10 nM; *p* = 0.69, *N* = 5, *n* = 10; 155.6% for SAG at 100 nM; *p* = 0.007, *N* = 4, *n* = 8; and 122.7% for SAG at 1 μM; *p* = 0.004, *N* = 7, *n* = 10; Wilcoxon test. **p < 0.01.

After establishing the role of SAG on ongoing network activity, we investigated whether it may affect GABAergic synaptic transmission. We performed whole-cell patch-clamp recording in acute hippocampal slices at P5-7 by measuring spontaneous GABA_A_ receptor-mediated postsynaptic currents (sGABA_A_-IPSCs) in CsCl-containing intrapipette solution (see “Materials and Methods” section). Figure 2A illustrates a typical experiment in which SAG (100 nM) produced an increase in sGABA_A_-IPSCs frequency within 10 min. Application of SAG in the presence of NBQX and D-APV increases sGABA_A_-IPSCs frequency, which returns to baseline after wash (Figures 2B,C). Conversely, we found that SAG did not affect the amplitude on sGABA_A_-IPSCs (Figure 2D). Furthermore, we observed a dose-dependent effect of SAG on sGABA_A_-IPSCs frequency with a threshold and maximal response at 100 nM (Figure 2E).

These data show that the Smo agonist SAG induces a specific dose-dependent increase in spontaneous GABAergic postsynaptic currents.

### SAG Deregulates the GABAergic/Glutamatergic Transmission Balance

**Figure 3:**
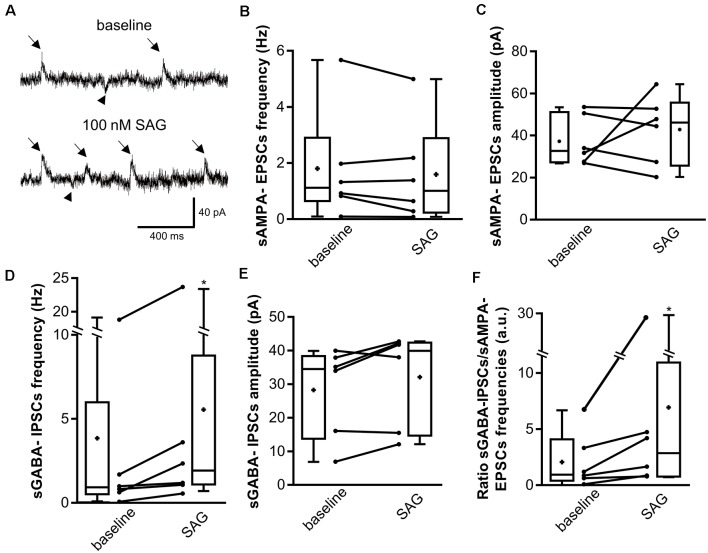
SAG regulates the excitatory/inhibitory spontaneous activity balance. **(A)** Representative traces of spontaneous AMPA excitatory post-synaptic currents (sAMPA-EPSCs, downward deflections, arrowhead) and GABAergic inhibitory post-synaptic currents (sGABA-IPSCs, upward deflections, arrows) during baseline and SAG application (100 nM). **(B–E)** Box plots and individual data points show median sAMPA-EPSCs **(B,C)** and sGABA-IPSCs **(D,E)** frequency **(B,D)** and amplitude **(C,E)** during baseline and SAG application. Median values: For sAMPA frequency: 1.12 Hz for baseline vs. 1.01 Hz during SAG; *p* = 0.31, *N* = 6 animals, *n* = 6 slices. For sAMPA amplitude: 32.9 pA for baseline vs. 46.16 pA during SAG; *p* = 0.99, *N* = 6, *n* = 6; Wilcoxon test. Median values: For sGABA frequency: 0.90 Hz for baseline vs. 1.77 Hz during SAG; *p* = 0.03, *N* = 6, *n* = 6. For sGABA amplitude: 34.5 pA for baseline vs. 39.9 pA during SAG; *p* = 0.15, *N* = 6, *n* = 6; Wilcoxon test. **(F)** Box plots and individual data points show ratio of sGABA-IPSCs over sAMPA-EPSCs frequencies. Median values: 1.03 a.u. for baseline, vs. 2.92 a.u. during SAG; *p* = 0.015, *N* = 6, *n* = 6; Wilcoxon test. *p < 0.05.

Since SAG affects the GABAergic synaptic transmission, we next sought to determine whether the GABAergic/glutamatergic transmission balance is altered by enhancing Shh-Smo signaling. We recorded hippocampal neurons from P5-7 rat slices using K-Gluconate intrapipette solution with voltage-clamp at −45 mV to measure simultaneously GABAergic and glutamatergic spontaneous activity (sGABA_A_-IPSCs and sAMPA-EPSCs, respectively). As observed in Figure 3A, SAG application does not change sAMPA-EPSCs (downward deflections in Figure 3A) frequency (Figures 3A,B) nor its amplitude (Figures 3A,C), but increases sGABA_A_-IPSCs (upward deflections in Figure 3A) frequency (Figures 3A,D) without modifying its amplitude (Figures 3A,E), mimicking the effect observed on sGABA-IPSCs under CsCl conditions (Figures 2D,E). These findings lead to a significant increase in the sGABA_A_-IPSCs over sAMPA-EPSCs frequency ratio during SAG application when compared to the control baseline (Figure 3F).

Altogether, these results show that acute Smo activation does not affect spontaneous glutamatergic transmission while enhances GABAergic transmission, thus, leads to a GABAergic/glutamatergic transmission imbalance in the newborn rat hippocampus.

### SAG Requires an Intracellular Ca^2+^ Signal to Increase Spontaneous GABAergic Activity

**Figure 4:**
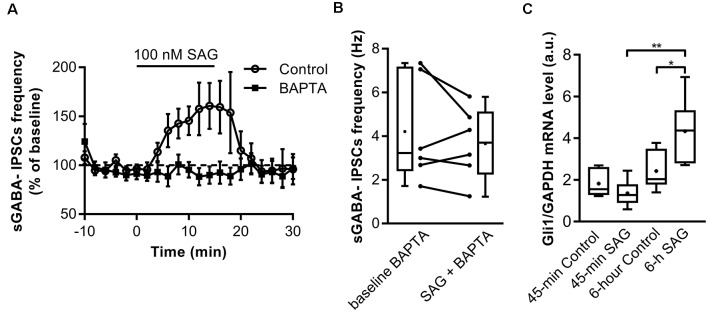
SAG regulates GABA spontaneous activity through non-canonical signaling. **(A)** Time course of mean sGABA-IPSCs frequency during SAG application (100 nM) normalized to baseline period, in control conditions (circles) or with intracellular Ca^2+^-chelator BAPTA (squares, 10 nM). **(B)** Box plots and individual data points show median sGABA-IPSCs frequency during baseline or BAPTA + SAG application. Median values: 3.21 Hz for baseline BAPTA, vs. 3.71 Hz during SAG + BAPTA; *p* = 0.56, *N* = 5 animals, *n* = 6 slices; Wilcoxon test. **(C)** Transcription factor Gli1 mRNA level during short (45 min) or long (6 h) incubation with 100 nM SAG in hippocampal slices measured by qRT-PCR. Median values: 1.55 a.u. in 45 min control condition, *n* = 6; 1.27 a.u. for 45 min SAG, *n* = 6; 2.035 a.u. in 6 h-control condition, *n* = 6 and 4.36 a.u. for 6 h SAG, *n* = 6; *p* = 0.18 for 45 min control vs. 45 min SAG; *p* = 0.02 for 6 h-control vs. 6 h SAG; *p* = 0.002 for 45 min SAG vs. 6 h SAG; Mann–Whitney *U* test. *p < 0.05, **p < 0.01.

We next investigated the mechanisms triggered by SAG to potentiate spontaneous GABAergic activity. Previous studies have shown that Shh signaling through Smo increases spontaneous Ca^2+^ transient frequency in embryonic spinal cord neurons (Belgacem and Borodinsky, 2011; Feng et al., 2016). We assessed the contribution of intracellular Ca^2+^ to SAG-induced increase in spontaneous GABAergic synaptic transmission. To this end, we performed whole-cell dialysis of recorded neurons with the Ca^2+^ chelator BAPTA (10 mM) and found that BAPTA abolished the SAG-induced increase in sGABA_A_-IPSCs frequency (Figures 4A,B).

The fast action of SAG on the sGABA_A_-IPSCs frequency and the dependence on intracellular Ca^2+^, suggest that enhancing SAG signaling activates a non-canonical Smo mediated pathway. Canonical Shh-Smo signaling leads to an increased expression of its downstream target gene Gli1 (Ruiz i Altaba, 1998; Jacob and Lum, 2007), whereas non-canonical pathway inhibits Gli1 expression in the developing spinal cord (Belgacem and Borodinsky, 2015). To further investigate whether the acute response to Smo activation triggers a non-canonical signaling pathway, Gli1 expression was measured using qRT-PCR after incubating the hippocampal slices for 45-min or 6 h with 100 nM SAG. The results show that 45-min SAG treatment does not change Gli1 transcription factor mRNA levels, while 6 h incubation induces an increase in Gli1 mRNA transcripts (Figure 4C).

Overall, these data show that SAG-induced-increase in sGABA_A_-IPSCs frequency is dependent on postsynaptic Ca^2+^ signaling probably through non-canonical Shh-Smo signaling pathway.

### BDNF-TrkB Signaling Is Required for SAG to Potentiate GABAergic Activity

**Figure 5:**
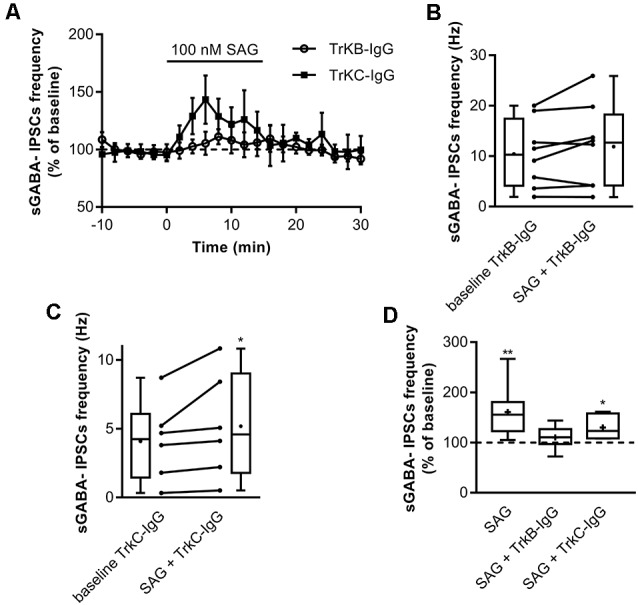
SAG recruits BDNF-TrkB signaling to potentiate GABAergic activity. **(A)** Time course of mean sGABA-IPSCs frequency during SAG (100 nM) application normalized to baseline period, with BDNF-scavenger TrkB-IgG (circles) or NT3-scavenger TrkC-IgG (squares) preincubation. **(B)** Box plots and individual data points show sGABA-IPSCs frequency after 1 h of preincubation with TrkB-IgG during baseline (baseline TrkB-IgG) and SAG application (SAG + TrkB-IgG). Median values: 10.3 Hz for baseline, vs. 12.7 Hz for SAG + TrkB IgG; *p* = 0.19, *N* = 3, *n* = 8; Wilcoxon test. **(C)** Box plots and individual data points show sGABA-IPSCs frequency after 1 h of preincubation with TrkC-IgG during baseline (baseline TrkC-IgG) and SAG application (SAG + TrkC-IgG). Median values: 4.24 Hz for baseline, vs. 4.59 Hz for SAG + TrkC-IgG; *p* = 0.03, *N* = 3, *n* = 6; Wilcoxon test. **(D)** Box plots show median sGABA-IPSCs frequency during SAG application, SAG + TrkB-IgG preincubation or SAG + TrkC-IgG preincubation, normalized to baseline period. Median values: 155.6% for SAG alone; *p* = 0.007, *N* = 4, *n* = 8; 110.4% for SAG + TrkB-IgG; *p* = 0.19, *N* = 3, *n* = 8; and 123.3% for SAG + TrkC-IgG; *p* = 0.03, *N* = 3, *n* = 6; Wilcoxon test. *p < 0.05, **p < 0.01.

Previous findings have shown that activation of Shh signaling increases BDNF levels (Dai et al., 2011; Radzikinas et al., 2011; Bond et al., 2013; He et al., 2016) and given that regulated release of BDNF increases spontaneous GABAergic synaptic activity *via* its high-affinity receptor TrkB (Gubellini et al., 2005; Kuczewski et al., 2008a, 2011), we assessed whether the effect of SAG on GABAergic activity requires the activation of the BDNF-TrkB signaling pathway. To test this hypothesis, hippocampal slices were incubated with the BDNF-scavenger TrkB-IgG (1 μg/ml) or with the NT3-scavenger TrkC-IgG (1 μg/ml) as control. We found that TrkB-IgG prevented the SAG-induced increase in sGABA_A_-IPSCs frequency (Figures 5A,B,D), while TrkC-IgG did not block the effect of SAG on sGABA_A_-IPSCs frequency (Figures 5A,C,D).

These data suggest that the Smo agonist SAG enhances spontaneous GABAergic synaptic transmission in a BDNF-TrkB-dependent manner.

### Shh Activates the BDNF-TrkB Signaling Pathway

**Figure 6:**
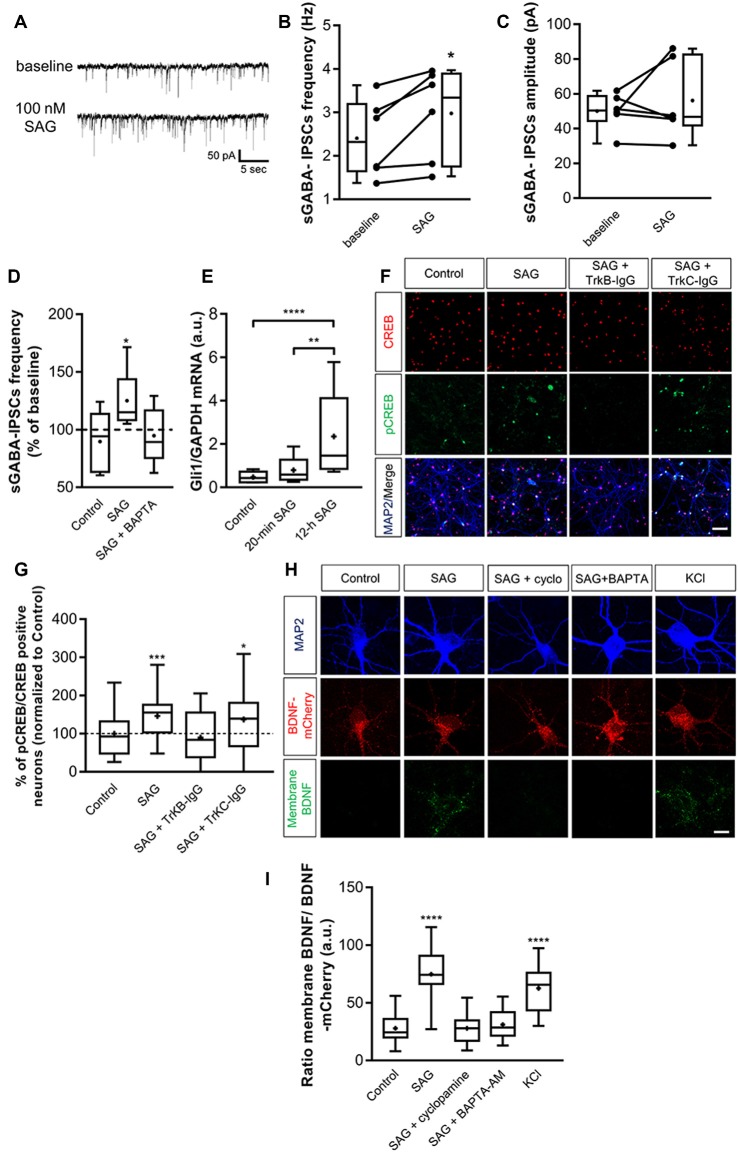
Smo pathway induces BDNF release from hippocampal neurons grown *in vitro*. **(A)** Representative traces of spontaneous GABAergic inhibitory post-synaptic currents (sGABA-IPSCs) during control baseline or SAG application (100 nM) in cultured hippocampal neurons at 14 DIV. **(B,C)** Box plots and individual data points show sGABA-IPSCs frequency **(B)** and amplitude **(C)** during baseline and SAG application. Median values: For sGABA-IPSCs frequency: 2.31 Hz for baseline; and 3.32 Hz during SAG; *p* = 0.015, *N* = 4 cultures, *n* = 6 cells; Wilcoxon test. For sGABA-IPSCs amplitude: 50.3 pA for baseline and 46.66 pA during SAG; *p* = 0.62, *N* = 4, *n* = 6; Wilcoxon test. **(D)** Box plots show median sGABA-IPSCs frequency normalized to baseline period, during application of carrier only (control), 100 nM SAG alone (SAG), or SAG with intracellular Ca^2+^-chelator BAPTA (SAG + BAPTA). Median values: 94.2% for Control; *p* = 0.21, *n* = 5; 115% during SAG; *p* = 0.015, *n* = 6; 89.27% during SAG + BAPTA; *p* = 0.62, *n* = 5; Wilcoxon test. **(E)** Transcription factor Gli1 mRNA level during short (20 min) or long (12 h) incubation with 100 nM SAG in hippocampal neuron culture at 14 DIV measured by qRT-PCR. Median values: 0.42 a.u. for Control, *N* = 8; 0.58 a.u. for 20-min SAG, *N* = 8; and 1.46 a.u. for 12-h SAG, *N* = 11; *p* = 0.19 for Control vs. 20-min SAG; *p* = 0.0001 for Control vs. 12-h SAG; *p* = 0.006 for 20-min SAG vs. 12 h-SAG. *p < 0.05, **p < 0.01, ***p < 0.005, ****p < 0.001; Mann–Whitney *U* test. **(F)** Cultured hippocampal neurons immunostained for CREB (red, top), pCREB (green, middle) in control conditions (control), or treated with 100 nM SAG alone (10 min; SAG), SAG plus TrkB-IgG preincubation (SAG + TrkB-IgG), or with SAG plus TrkC-IgG preincubation (SAG + TrkC-IgG). Down, merged images with MAP2 staining (blue). Scale bar: 50 μm. **(G)** Box plots of pCREB on CREB immunostained neurons in different conditions, normalized to control for each culture. Median values: 92.65% for Control, *N* = 5 cultures, *n* = 34 fields; 155.2% for SAG, *N* = 5, *n* = 41; 83.79% for SAG + TrkB-IgG, *N* = 5, n = 35; and 139.1% for SAG + TrkC-IgG, *N* = 4, *n* = 25; *p* = 0.0004 for Control vs. SAG; *p* = 0.41 for Control vs. SAG + TrkB-IgG; *p* = 0.03 for Control vs. SAG + TrkC-IgG; Mann–Whitney *U* test. **(H)** SAG induces BDNF release. BDNF-mCherry transfected neurons (red) are live immunostained with antibody against mCherry (green) in non-permeable condition and treated during 10 min with KCl (50 mM) or SAG (100 nM) with or without cyclopamine (2 μM) or BAPTA (10 μM). Neurons are immunostained after fixation with an antibody against MAP2 (blue). Scale bar: 10 μm. **(I)** Box plots show median fluorescence intensity ratio of released membrane-bound BDNF on total BDNF-mCherry in different culture conditions. Median values: 24.46 a.u. for control, N = 3 cultures, n = 32 cells; 74.25 a.u. for SAG, *N* = 3 cultures, *n* = 31 cells; 27.94 a.u. for SAG + cyclopamine, *N* = 3, *n* = 37; 28.56 a.u. for SAG + BAPTA-AM, *N* = 3, *n* = 25; and 65.7 a.u. for KCl, *N* = 3, *n* = 30; *p* = 0.0001 for control vs. SAG; *p* = 0.85 for control vs. SAG + cyclopamine; *p* = 0.28 for control vs. SAG + BAPTA-AM; *p* = 0.0001 for control vs. KCl; Mann–Whitney *U* test. *p < 0.05, **p < 0.01, ***p < 0.005, ****p < 0.001.

To confirm that SAG recruits the BDNF signaling, we decided to use an *in vitro* model of primary hippocampal neuron cultures. We first performed whole-cell patch-clamp recordings on primary hippocampal neuron cultures to measure spontaneous GABAergic synaptic activity at 14 days *in vitro* (DIV). Following our previous results obtained on hippocampal slices, 100 nM SAG increases the sGABA_A_-IPSCs frequency (Figures 6A,B) and does not affect the amplitude of sGABA_A_-IPSCs (Figures 6A,C). As observed in hippocampal slices, BAPTA prevents the increase in sGABA_A_-IPSCs frequency produced by SAG application (Figure 6D). Altogether, these results confirm those obtained using acute hippocampal slices and validate the use of hippocampal cell cultures to further investigate the functional link between SAG and BDNF-TrkB signaling. To confirm that acute stimulation of hippocampal neurons with SAG does not activate a canonical pathway, we assessed Gli1 expression. Results show that 20-min SAG treatment in hippocampal neuron cultures does not change Gli1 transcription factor mRNA levels, while 12 h incubation induces a 3-fold increase in Gli1 transcripts (Figure 6E). To assess the effect of SAG (10 min incubation at 100 nM) on the BDNF-TrkB signaling pathway, we measured the levels of the phosphorylated form of cAMP-response element-binding protein (CREB), a direct downstream target of BDNF-TrkB (Ghosh et al., 1994). The ratio between pCREB and CREB immunopositive neurons was quantified and expressed as a percentage of the control condition for each culture (Figures 6F,G). SAG induces a significant increase in the pCREB/CREB ratio, which is prevented by pre-incubation with the TrkB-IgG scavenger and not by the TrkC-IgG scavenger (Figures 6F,G). Finally, to investigate the ability of SAG to induce BDNF secretion, mCherry-tagged BDNF was overexpressed in cultured hippocampal neurons (Kuczewski et al., 2008b; Porcher et al., 2011). We compared levels of BDNF-mCherry bound to the cell membrane of transfected neurons with an antibody directed against mCherry (see “Materials and Methods” section) in cultures subjected to different conditions. In transfected neurons, 10 min incubation with 50 mM KCl produced green staining surrounding the BDNF-mCherry expressing neurons, reflecting the release of BDNF (Figure 6H, bottom panel). In contrast, in the control condition, the cell surface-bound of BDNF-mCherry was barely detectable (Figures 6H,I). We found that incubation with SAG (100 nM; 10 min) produced a significant increase in membrane-bound BDNF-mCherry (Figures 6H,I). This effect was prevented by the addition of 2 μM cyclopamine or 10 μM BAPTA-AM (Figures 6H,I).

Altogether, our findings suggest that Shh-Smo signaling upregulates the secretion of BDNF and consequently the activation of the BDNF-TrkB signaling pathway.

## Discussion

Shh is a prominent neurotrophic factor that acts at multiple levels during central nervous system development from embryonic through postnatal stages, and perturbations in its activity lead to major neurodevelopmental disorders (Currier et al., 2012; Boyd et al., 2015; Halepoto et al., 2015; Feng et al., 2016; Patel et al., 2017; Kumar et al., 2019; Sasai et al., 2019). Here, we demonstrate in both hippocampal slices and neuronal cultures that the Smo agonist SAG modulates spontaneous network activity and GABAergic synaptic plasticity in the maturing rodent hippocampus through the secretion and signaling of BDNF and an intracellular Ca^2+^-dependent mechanism.

We found that in the postnatal hippocampus Smo activation leads to a long-lasting increase in GDP frequency that occurs only during the first postnatal week, suggesting the existence of a specific temporal window for Shh-Smo signaling modulation of spontaneous network activity. However, whether Shh-Smo signaling regulates sGABA-IPSCs in the mature hippocampus (i.e., after P15) will require further study. Although, we did not observe a progressive increase in Shh levels as observed using immunoblots (Rivell et al., 2019), we confirmed by ELISA that Shh protein is expressed in the postnatal hippocampus. Interestingly, this period corresponds to a critical developmental phase during which synaptogenesis and network formation take place (Griguoli and Cherubini, 2017). Further, we showed that Smo and Ptch1 transcript levels are downregulated at the end of the first postnatal week, suggesting that the expression of Shh co-receptors may participate in the process of establishing hippocampal neuronal circuits. Similarly, a spatiotemporal expression of Shh components has been recently described in the developing human cerebral cortex (Memi et al., 2018).

It is well known that the ongoing polysynaptic events are dependent on synchronized GABAergic and glutamatergic synaptic transmission (Ben-Ari et al., 1989) and are thought to be important for the generation of neuronal networks, serving as a coincidence detector to reinforce synapses from neurons spiking together (Kasyanov et al., 2004; Mohajerani et al., 2007; Griguoli and Cherubini, 2017). The higher sensitivity to SAG observed on polysynaptic GDP events as compared to monosynaptic sGABA-IPSCs activity may reflect the cumulative effects of SAG in network-driven activities. At the cellular level, our results indicate that SAG enhances GABAergic synaptic transmission without affecting the glutamatergic component. This selective action of SAG on GABAergic synaptic currents reveals one plausible mechanism underlying Shh-Smo modulation of GDP frequency in the maturing hippocampus. Whether SAG requires a GABAergic excitatory response in pyramidal cells remains to be elucidated but we can hypothesize that these effects are unlikely to be related to the depolarizing action of GABA because the SAG-induced increase in GABAergic activity required a postsynaptic rise in calcium as the effect was prevented when the recorded neurons were loaded with 10 mM BAPTA. Additionally, the efficacy of SAG to potentiate GABAergic activity was observed under conditions where GABA is depolarizing (CsCl solution) as well as under conditions where GABA is hyperpolarizing (K-Gluconate solution). We find that this increase in GABAergic inputs leads to a GABAergic/glutamatergic imbalance. Alterations in excitatory/inhibitory balance and GDP dynamics have been involved in the onset of neurodevelopmental disorders (Griguoli and Cherubini, 2017), including autistic-like behavior phenotype (Tyzio et al., 2014) or increased seizure susceptibility (Chiu et al., 2008). Interestingly, previous studies reported abnormally increased serum levels of Shh in children with Autism Spectrum Disorder (Al-Ayadhi, 2012; Halepoto et al., 2015), thus suggesting a possible causal link between alterations in Shh signaling and neurodevelopmental disorders (Kumar et al., 2019). Other trophic factors are known to modulate spontaneous ongoing network activity such as the chemokine stromal cell-derived factor-1-alpha (SDF-1α; Kasyanov et al., 2006), BDNF (Mohajerani et al., 2007) and the hormone leptin (Guimond et al., 2014; Dumon et al., 2019). Both BDNF and leptin increase spontaneous GABAergic postsynaptic current frequency through the extracellular signal-related kinase (ERK) pathway. In contrast, Shh inhibits ERK in an oxidative stress model of cultured cortical neurons to prevent apoptosis (Dai et al., 2012). The identification of downstream effectors and their spatiotemporal regulation in the Shh-mediated enhancement of GABAergic transmission in the early postnatal hippocampus requires further investigation.

Activation of Smo-dependent canonical Shh signaling leads to the recruitment of the Gli-family transcription factors (Briscoe and Thérond, 2013). However, this pathway is transcription-dependent, thus, it is not likely to mediate acute changes in synaptic physiology. On the other hand, non-canonical Smo-dependent signaling involves a fast response that often relies on kinases such as PKA, Src family kinases and second messengers such as Ca^2+^ and cAMP (Charron et al., 2003; Riobo et al., 2006; Belgacem and Borodinsky, 2011, 2015; Peng et al., 2018). In this study, we found that SAG modulation of spontaneous GABAergic synaptic activity at the cellular and network levels occurs within minutes, suggesting the involvement of such non-canonical Shh-Smo signaling in this process. The non-canonical Shh signaling in the embryonic spinal cord is dependent on inositol triphosphate (IP3) second messenger and Ca^2+^ activity through intracellular Ca^2+^ stores and Ca^2+^ influx (Belgacem and Borodinsky, 2011, 2015). These second messengers are involved in the modulation of developmental forms of GABAergic synaptic plasticity (Ben-Ari et al., 2007; Guimond et al., 2014; Dumon et al., 2019). Also, two recent studies suggested that non-canonical Shh signaling is likely to induce Ca^2+^ oscillations in hippocampal glial cells through the activation of ATP-permeable channels (Adachi et al., 2019) and may produce in hippocampal neuronal cultures an intracellular Ca^2+^ increase mediated by the activation of the NMDA receptor (Feng et al., 2016).

We show that SAG-induced potentiation of GABA_A_ post-synaptic currents is prevented by chelating intracellular Ca^2+^ in hippocampal neurons. This observation indicates that SAG modulates spontaneous GABAergic activity in the developing hippocampus through post-synaptic Ca^2+^ dynamics. Our experiments have been realized in the presence of ionotropic glutamate receptor antagonists, which exclude the possibility of Ca^2+^ entry through the NMDA receptors. Further, our study indicates that the trophic action of SAG was blocked by the scavenging BDNF. It is worth noting that BDNF is an important factor playing a crucial role in synaptic plasticity and network assembly (Lu, 2003; Kuczewski et al., 2009; Porcher et al., 2018). Moreover, it has been demonstrated that ongoing synaptic activity in the developing rodent hippocampus can trigger the post-synaptic release of BDNF through Ca^2+^ influx (Kuczewski et al., 2008a,b; Fiorentino et al., 2009). Within the hippocampus, BDNF is mainly expressed by principal glutamatergic neurons and released in an activity-dependent manner. Therefore, our findings support a model in which SAG promotes GABAergic synaptic activity through the secretion of BDNF by excitatory pyramidal cells in the immature hippocampus. This interplay between Shh-Smo signaling and BDNF is not well understood but studies realized in cortical neurons, sciatic and cavernous nerves indicated that Shh up-regulates BDNF secretion to promote regeneration of injured neurons (Hashimoto et al., 2004; Dai et al., 2012; Bond et al., 2013). In the lung epithelium, Shh has been reported to indirectly regulate the expression of BDNF through the inhibition of a BDNF repressor (Radzikinas et al., 2011).

In conclusion, our results shed a light on a new role for the morphogenic peptide Shh in the maturation and refinement of GABAergic synaptic transmission in the rodent postnatal hippocampus. We have shown that the non-canonical Smo signaling may likely involve the secretion of BDNF to trigger an increase of spontaneous GABAergic post-synaptic currents. Altogether, these results bring therefore new perspectives for the function of this versatile protein in shaping neuronal networks during brain maturation and its involvement in neurodevelopmental disorders.

## Data Availability Statement

The datasets generated for this study are available on request to the corresponding author.

## Ethics Statement

The animal study was reviewed and approved by INSERM animal welfare through the local committee and the European Communities Council Directive of 24 November 1986 (86/609/EEC).

## Author Contributions

YHB, QD, and CP planned the experiments. QD, MH, YB, DD, CP, YHB, AM, MK, and J-LG performed the experiments and analyzed the data. QD, YHB, and CP wrote the manuscript.

## Conflict of Interest

The authors declare that the research was conducted in the absence of any commercial or financial relationships that could be construed as a potential conflict of interest.

## Footnotes

^1^http://rsb.info.nih.gov/ij/
